# Supplementary material for: Crystal structures of human ETB receptor provide mechanistic insight into receptor activation and partial activation
Source: Nat Commun. 2018 Nov 9;9:4711. doi: 10.1038/s41467-018-07094-0 (PMC6226434; doi:10.1038/s41467-018-07094-0)
Supplement: Supplementary file 1 — Supplementary Information [file 41467_2018_7094_MOESM1_ESM.pdf]

Crystal structures of human ET_B receptor provide mechanistic insight into receptor activation and partial activation

W. Shihoya, T. Izume et al.

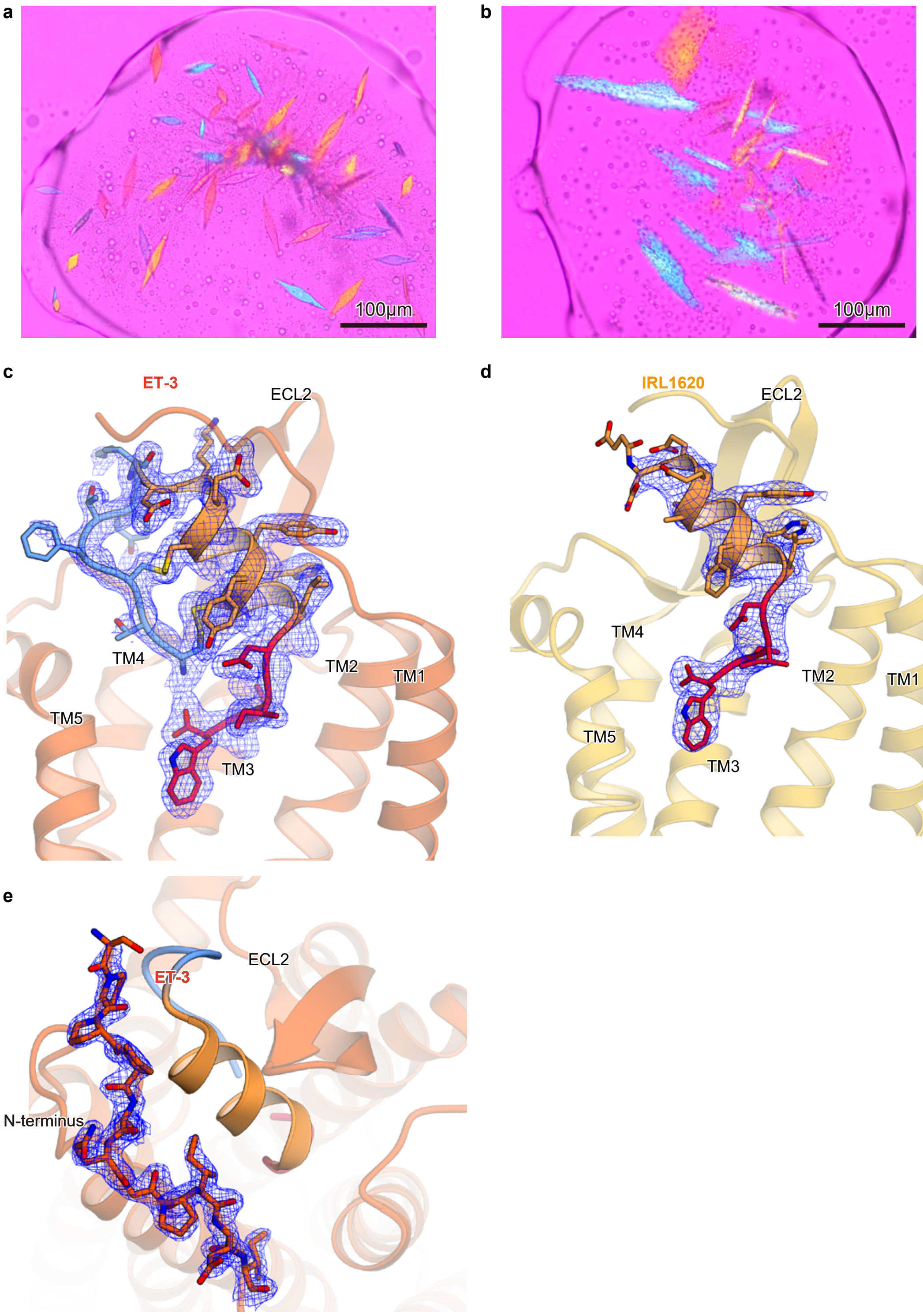

Supplementary Figure 1 | Crystals and electron densities.

a, **b**, Crystals of the ET_B receptors in complex with ET-3 (**a**) and IRL1620 (**b**). **c**, **d**, $F_o - F_c$ omit maps for ET-3 (**c**) and IRL1620 (**d**), contoured at 2.0σ. It should be noted that D8, E9, and N-terminal succinyl group of IRL1620 are not well resolved in the electron density. **e**, $2F_o - F_c$ map of the N-terminus in the ET-3-bound structure, contoured at 2.5σ.

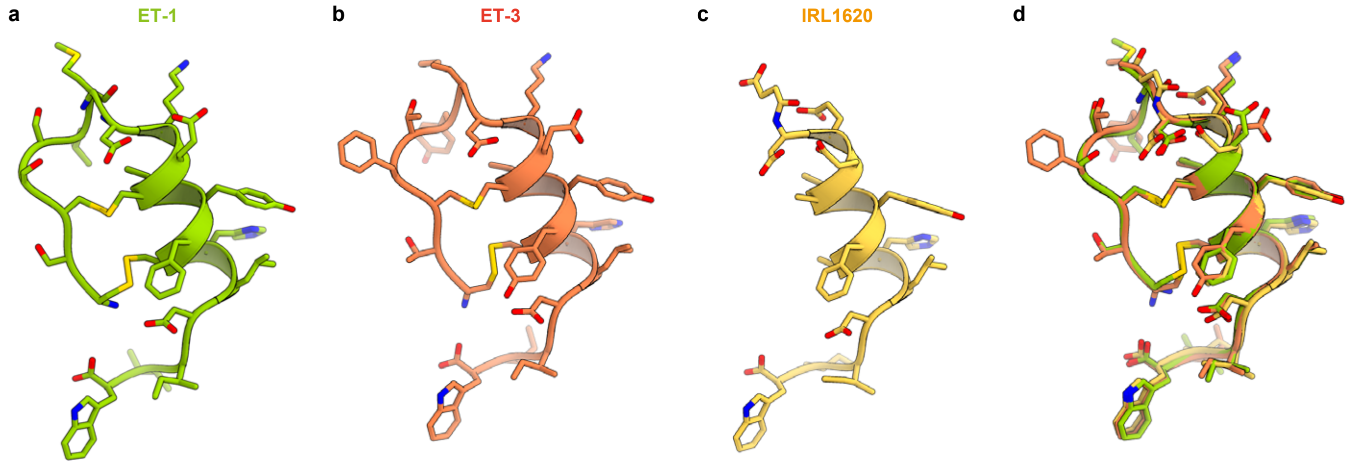

Supplementary Figure 2 | Comparison of agonist structures.

a-**c**, Structures of ET-1 (**a**), ET-3 (**b**), and IRL1620 (**c**) in the complex structures, coloured pink, orange-red, and orange, respectively. The agonists are shown as ribbon and stick models. **d**, Superimposition of the agonist structures.

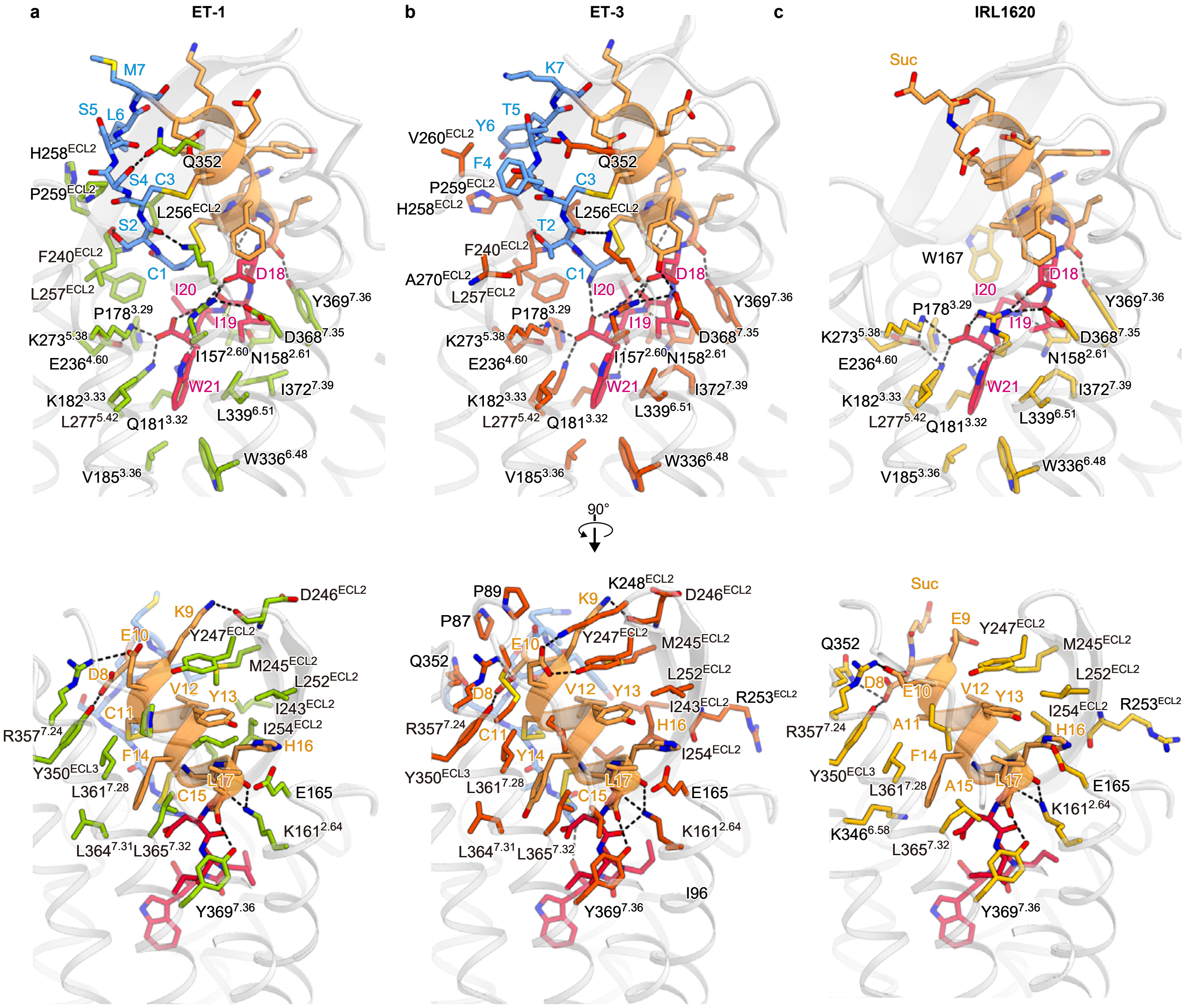

Supplementary Figure 3 | Comparison of receptor interactions of agonist peptides.

a-**c**, Comparison of the receptor binding interactions of ET-1 (**a**), ET-3 (**b**), and IRL1620 (**c**). The upper panels show the interactions of the N-terminal and C-terminal regions, and the lower panels show those of the α-helical regions. The agonists are shown as ribbon and stick models, coloured as in Figs. 2b and 3b. The structures of the receptors are shown as silver ribbons. The residues involved in the agonist binding are shown as sticks, coloured as in Supplementary Figure 2. We observed minor difference in the binding modes of ET-1 and ET-3. Three consecutive prolines (P88, P89, and P90) extend over the N-terminal region of ET-3 and form van der Waals interactions. In ET-1, E10 forms a salt bridge with R357$^{7.24}$, whereas in ET-3, E10 forms hydrogen-bonding interactions with Y247^{ECL2} and K248^{ECL2}. F4 of ET-3 forms a van der Waals interaction with Q352^{ECL3}. The N-terminal amide and the C-terminal carboxylate of ET-3 form a direct hydrogen bond. We also observed differences in the binding modes of the full agonist ET-3 and the partial agonist IRL1620. Due to the truncation of the N-terminal region, IRL1620 completely lacks the interactions via this region. K9 is replaced with glutamic acid (E9) in IRL1620, and interactions at the N-terminal end of the α-helical region are slightly diverged.

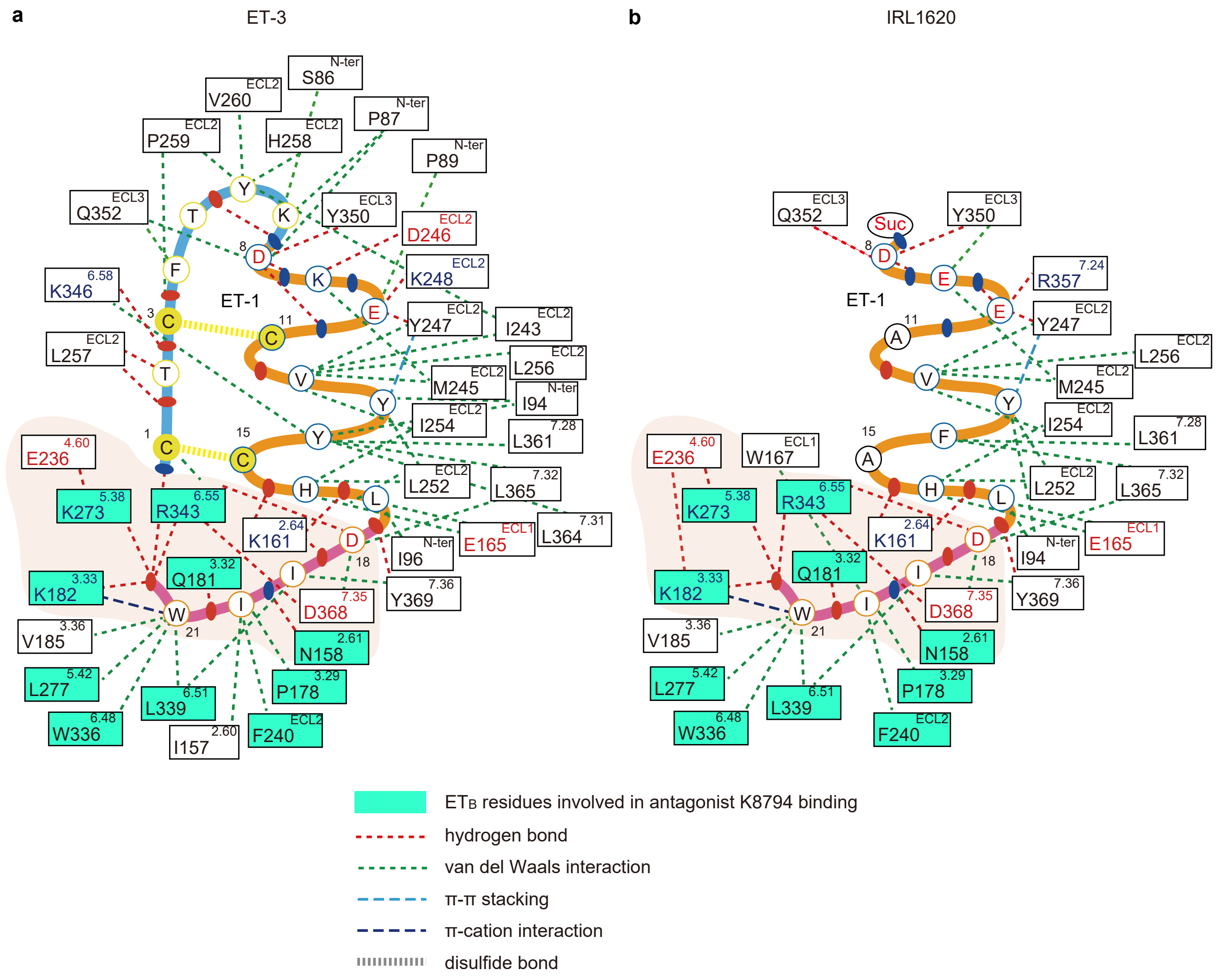

Supplementary Figure 4 | Binding plots of ET-3 and IRL1620

a, **b**, Schematic drawing of the binding interactions of ET-3 (**a**) and IRL1620 (**b**). The residues shown here are within a radius of 4 Å around the ligand in the crystal structure. Amino-acid residues of ET-3 and IRL1620 are represented by capital letters enclosed within circles. Blue and red ovals indicate main chain amide, and carbonyl and carboxyl groups of ET-3 and IRL1620, respectively. All residues of the ET_B receptor involved in the interactions are indicated by large boxes and amino-acid letters, and the residues involved in bosentan and K8794 binding are coloured turquoise. The types of interaction are indicated with dotted lines.

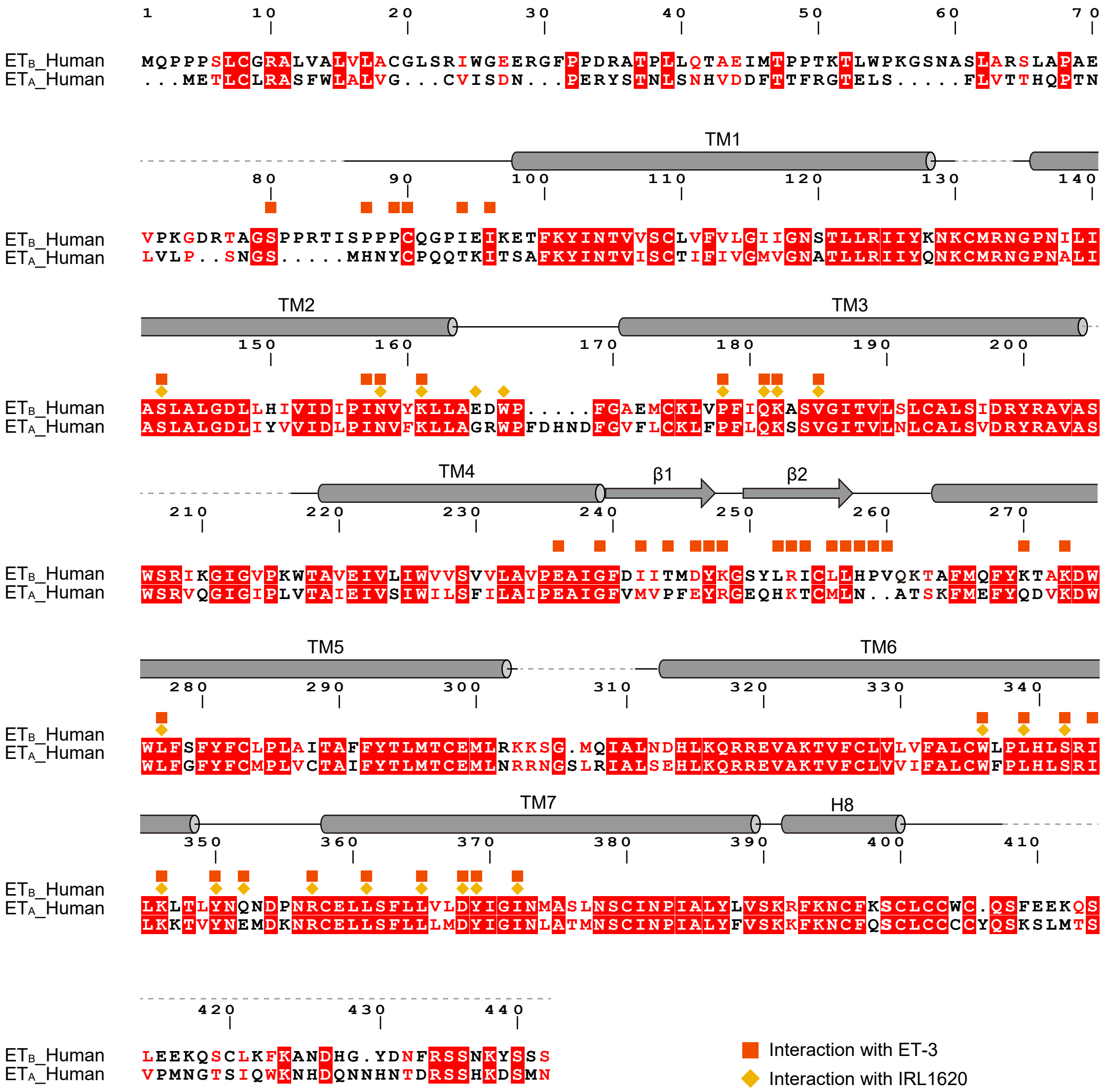

Supplementary Figure 5 | Alignment of human ETA and ETB receptor sequences.

Alignment of the amino acid sequences of the human ETB receptor (UniProt ID: P24530) and human ETA receptor (P25101). Secondary structure elements for α-helices and β-strands are indicated by cylinders and arrows, respectively. Conservation of the residues between ET_A and ET_B is indicated as follows: red panels for completely conserved; red letters for partially conserved; and black letters for not conserved. The residues involved in the ET-3 and IRL1620 binding are indicated with squares and diamonds, respectively.

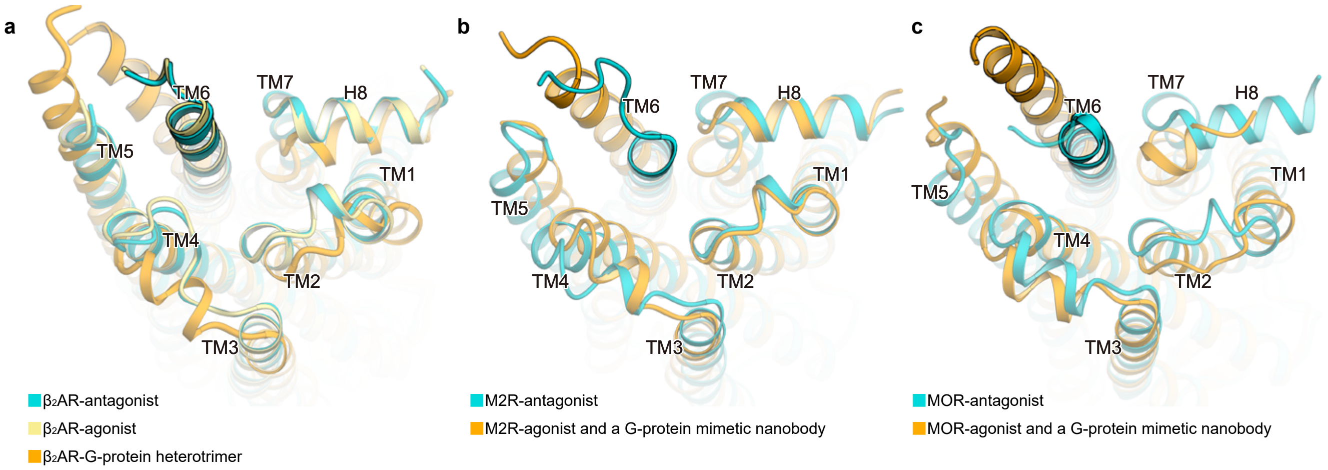

Supplementary Figure 6 | Comparison of the intracellular sides of class A GPCRs.

a, Structures of β_2 adrenergic receptor in complex with an antagonist (PDB 2RH1), an agonist (PDB 3PDS), and G-protein heterotrimer (PDB 3SN6), coloured turquoise, khaki, and orange, respectively. **b**, Structures of M2 receptor bound to an antagonist (PDB 3UON), and in complex with an agonist and G-protein mimetic nanobody (PDB 4MQS), coloured turquoise and orange, respectively. **c**, Structures of μ-opioid receptor bound to an antagonist (PDB 4DKL), and in complex with an agonist and a G-protein mimetic nanobody (PDB 5C1M), coloured turquoise and orange, respectively. Upon G-protein binding, the intracellular side of TM7 moves inward by 1 to 2 Å, and that of TM6 moves outward by 10 to 14 Å. These movements are a common structural feature among the class A GPCRs. The intracellular side of TM6 is not opened up in the agonist-bound structure of the β_2 adrenergic receptor (PDB 3PDS). This outward movement of TM6 is highly dependent on the G-protein binding, and agonist binding only induces structural heterogeneity on the intracellular side of TM6.

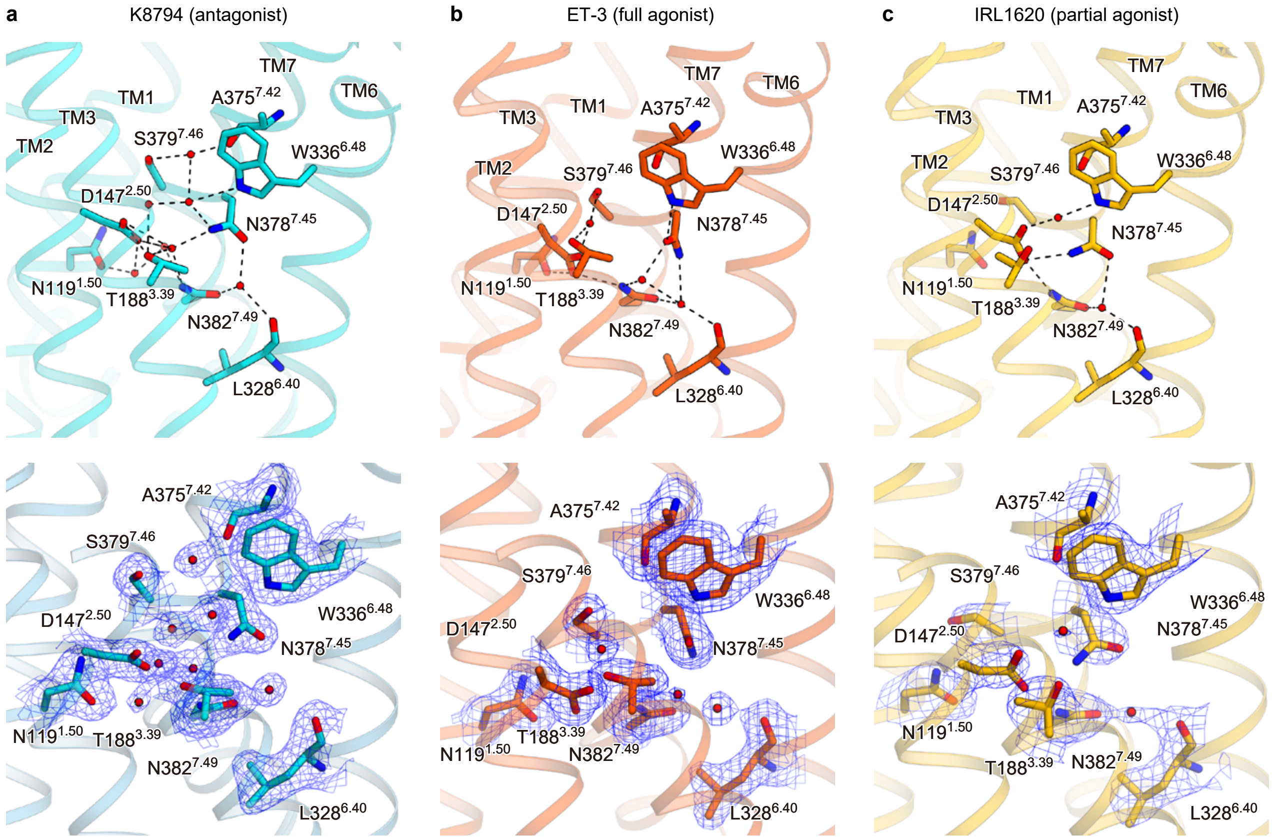

Supplementary Figure 7 | Water-mediated hydrogen-bonding network at the receptor core.

a-**c**, Hydrogen-bonding networks in the intermembrane regions of the K8794- (**a**), ET-3- (**b**), and IRL1620- (**c**) bound structures, coloured as in Fig. 5. The upper panels show the overall hydrogen-bonding interactions. Waters are shown as red spheres, and hydrogen bonding interactions are indicated by dashed lines. The lower panels show the $2F_o - F_c$ maps around D147$^{2.50}$ and W336$^{6.48}$, contoured at 1.0σ.

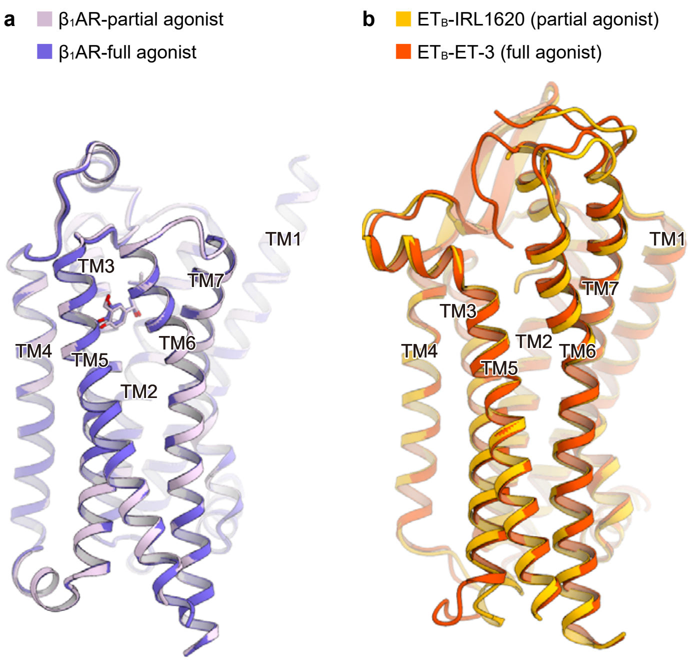

Supplementary Figure 8 | Comparison with β_1-adrenergic receptor structures.

a, Superimposition of the β_1-adrenergic receptor structures in complex with the full agonist isoprenaline (PDB 2Y03) and the partial agonist salbutamol (PDB 2Y04) (overall R.M.S.D of 0.25 Å for the Cα atoms). **b**, Superimposition of the ET_B structures in complex with the full agonist ET-3 and the partial agonist IRL1620 (overall R.M.S.D of 0.91 Å for the Cα atoms).

Supplementary Table 1 | The nucleotide sequence of the crystallization construct ET_B-Y5-T4L

ET_B-Y5-T4L	ATGAAGACTATCATCGCTCTCAGCTACATCTTCTGCCTGGTCTTCGCCGATTACAAGGATGACGACGATGCCATGGGTCA<br>GCCTGTCGGCGCGCCGGGCTTCCCGCCTGACAGGGCCACTCCGCTTTTGCAAACCGCAGAGATAATGACGCCACCCACTA<br>AGACCTTATGGCCCAAGGGTGAAAACCTGTATTTTCAGGGCGGTGGGCTAGCACCTGCGGAGGTGCCTAAAGGAGACAG<br>GACGGCAGGATCTCCGCCACGCACCATCTCCCCTCCCCCGTGCCAAGGACCCATCGAGATCAAGGAGACTTTCAAATACA<br>TCAACACGGTTGTGTCCTGCCTTGTGTTCGTGCTGGGGATCATCGGGAACTCCACACTTCTGTACATTATCTACAAGAACA<br>AGTGCATGCGAAACGGTCCCAATATCTTGATCGCCAGCTTGGCTCTGGGAGACCTGCTGCACATCGTCATTGCCATCCCT<br>ATCAATGTCTACAAGCTGCTGGCAGAGGACTGGCCATTTGGAGCTGAGATGTGTAAGCTGGTGCCTTTCATACAGAAAGC<br>CTCCGTGGGAATCACTGTGCTGAGTCTATGTGCTCTGAGTATTGACAGATATCGAGCTGTTGCTTCTTGGAGTAGAATTAA<br>AGGAATTGGGGTTCCAAAATGGACAGCAGTAGAAATTGTTTTGATTTGGGTGGTCTCTGTGGTTCTGGCTGTCCCTGAAG<br>CCATAGGTTTTGATATAATTACGATGGACTACAAAGGAAGTTATCTGCGAATCTGCTTGCTTCATCCCGTTCAGAAGACA<br>GCTTTCATGCAGTTTTACGCGACAGCAAAAGATTGGTGGCTGTTCAGTTTCTATTTCTGCTTGCCATTGGCCATCACTGCA<br>TTTTTTTATACACTAATGACCTGTGAAATGTTGAGAAAGAACATCTTCGAGATGCTGCGCATCGACGAAGGCCTGCGTCT<br>CAAGATTTACAAGAATACCGAAGGTTATTACACGATTGGCATCGGCCACCTCCTGACAAAGAGCCCATCACTCAACGCTG<br>CCAAGTCTGAACTGGACAAAGCCATTGGTCGCAACACCAACGGTGTCATTACAAAGGACGAGGCGGAGAAACTCTTCAA<br>CCAAGATGTAGATGCGGCTGTCCGTGGCATCCTGCGTAATGCCAAGTTGAAGCCCGTGTATGACTCCCTTGATGCTGTTC<br>GCCGTGCAGCCTTGATCAACATGGTTTTCCAAATGGGTGAGACCGGAGTGGCTGGTTTTACGAACTCCCTGCGCATGCTC<br>CAGCAGAAGCGCTGGGACGAGGCCGCAGTGAATTTGGCTAAATCTCGCTGGTACAATCAGACACCTAACCGTGCCAAGC<br>GTGTCATCACTACCTTCCGTACTGGAACTTGGGACGCTTACTTAAATGATCACCTTAAGCAGAGACGGGAAGTGGCCAAA<br>ACCGTCTTTTGCCTGGTCCTTGTCTTTGCCCTCTGCTGGCTTCCCCTTCACCTCGCCAGGATTCTGAAGCTCACTCTTTATA<br>ATCAGAATGATCCCAATAGATGTGAACTTTTGAGCTTTCTGTTGGTATTGGACTATATTGGTATCAACATGGCTTCACTGA<br>ATTCCTGCGCTAACCCAATTGCTCTGTATTTGGTGAGCAAAAGATTCAAAAACGCCTTTAAGTCAGCCTTATGCTGCTGG<br>GCCCAGTCA

Supplementary Table 2 | The nucleotide sequences of ET_B used in functional analysis

Flag-ET_B-WT<br>in pCAG vector	ATGCAGCCTCCTCCTAGCCTTTGTGGCAGAGCACTGGTGGCTCTGGTGCTGGCTTGTGGCCTGTCTAGAATCTGGGGCGA<br>AGAGAGAGGCTTCCCTCCTGATCGTGCTACCCCTCTGCTGCAGACAGCCGAGATCATGACCCCACCTACCAAGACACTGT<br>GGCCCAAGGGCGATTACAAGGATGACGACGATAAGCTGGCTCCTGCCGAAGTGCCTAAGGGCGATAGAACAGCCGGCTC<br>TCCACCTCGGACAATCAGCCCTCCACCTTGTCAGGGCCCCATCGAGATCAAAGAGACATTCAAGTACATCAACACCGTGG<br>TGTCCTGCCTGGTGTTCGTGCTGGGCATCATCGGCAATAGCACCCTGCTGCGGATCATCTACAAGAACAAGTGCATGCGG<br>AACGGCCCCAACATCCTGATCGCTTCTCTGGCCCTGGGAGATCTGCTGCACATCGTGATCGACATCCCCATCAACGTGTA<br>CAAGCTGCTGGCCGAGGATTGGCCTTTTGGCGCCGAGATGTGCAAGCTGGTGCCCTTCATCCAGAAAGCCAGCGTGGGC<br>ATCACAGTGCTGTCTCTGTGTGCCCTGAGCATCGACAGATACAGAGCCGTGGCCAGCTGGTCCCGGATCAAAGGCATTGG<br>AGTGCCAAAGTGGACCGCCGTGGAAATCGTGCTGATCTGGGTCGTGTCTGTGGTCCTGGCTGTGCCTGAGGCCATCGGCT<br>TCGACATCATCACCATGGACTACAAGGGCTCCTACCTGCGGATCTGCCTGCTGCACCCTGTGCAGAAAACCGCCTTCATG<br>CAGTTCTACAAGACCGCCAAGGATTGGTGGCTGTTCAGCTTCTACTTCTGCCTGCCTCTGGCCATCACCGCCTTTTTCTAC<br>ACCCTGATGACCTGCGAGATGCTGCGGAAGAAAAGCGGCATGCAGATCGCCCTGAACGACCACCTGAAGCAGAGAAGA<br>GAAGTGGCCAAGACCGTGTTTTGCCTCGTGCTGGTGTTTGCCCTGTGCTGGCTGCCTCTGCATCTGAGCAGAATCCTGAA<br>GCTGACCCTGTACAACCAGAACGACCCCAATAGATGCGAGCTGCTGAGCTTCCTGCTGGTCCTGGACTACATCGGCATCA<br>ACATGGCCAGCCTGAACAGCTGCATCAACCCTATCGCTCTGTACCTGGTGTCCAAGCGGTTCAAGAACTGCTTCAAGAGC<br>TGCCTGTGCTGCTGGTGCCAGAGCTTCGAGGAAAAGCAGAGCCTGGAAGAGAAGCAGAGCTGTCTGAAGTTCAAGGCCA<br>ACGACCACGGCTACGACAACTTCAGAAGCAGCAACAAGTACAGCAGCAGC
Flag-ET_B-Y5<br>in pCAG vector	ATGCAGCCTCCTCCTAGCCTTTGTGGCAGAGCACTGGTGGCTCTGGTGCTGGCTTGTGGCCTGTCTAGAATCTGGGGCGA<br>AGAGAGAGGCTTCCCTCCTGATCGTGCTACCCCTCTGCTGCAGACAGCCGAGATCATGACCCCACCTACCAAGACACTGT<br>GGCCCAAGGGCGATTACAAGGATGACGACGATAAGCTGGCTCCTGCCGAAGTGCCTAAGGGCGATAGAACAGCCGGCTC<br>TCCACCTCGGACAATCAGCCCTCCACCTTGTCAGGGCCCCATCGAGATCAAAGAGACATTCAAGTACATCAACACCGTGG<br>TGTCCTGCCTGGTGTTCGTGCTGGGCATCATCGGCAATAGCACCCTGCTGTACATCATCTACAAGAACAAGTGCATGCGG<br>AACGGCCCCAACATCCTGATCGCTTCTCTGGCCCTGGGAGATCTGCTGCACATCGTGATCGCCATTCCTATCAACGTGTAC<br>AAGCTGCTGGCCGAGGACTGGCCTTTTGGAGCCGAGATGTGCAAGCTGGTGCCCTTCATCCAGAAAGCCAGCGTGGGCA<br>TCACAGTGCTGTCTCTGTGTGCCCTGAGCATCGACAGATACAGAGCCGTGGCCAGCTGGTCCCGGATCAAAGGCATTGGA<br>GTGCCAAAGTGGACCGCCGTGGAAATCGTGCTGATCTGGGTCGTGTCTGTGGTCCTGGCTGTGCCTGAGGCCATCGGCTT<br>CGACATCATCACCATGGACTACAAGGGCTCCTACCTGCGGATCTGCCTGCTGCACCCTGTGCAGAAAACCGCCTTCATGC<br>AGTTCTACGCCACCGCCAAGGATTGGTGGCTGTTCAGCTTCTACTTCTGCCTGCCTCTGGCCATCACCGCCTTTTTCTACA<br>CCCTGATGACCTGCGAGATGCTGCGGAAGAAAAGCGGCATGCAGATCGCCCTGAACGACCACCTGAAGCAGAGAAGAG<br>AAGTGGCCAAGACCGTGTTTTGCCTCGTGCTGGTGTTTGCCCTGTGCTGGCTGCCTCTGCATCTGGCCAGGATTCTGAAGC<br>TGACCCTGTACAACCAGAACGACCCCAATAGATGCGAGCTGCTGAGCTTCCTGCTGGTCCTGGACTACATCGGCATCAAC<br>ATGGCCAGCCTGAACAGCTGCGCCAATCCTATCGCTCTGTACCTGGTGTCCAAGCGGTTCAAGAACTGCTTCAAGAGCTG<br>CCTGTGCTGCTGGTGCCAGAGCTTCGAGGAAAAGCAGAGCCTGGAAGAGAAGCAGAGCTGTCTGAAGTTCAAGGCCAAC<br>GACCACGGCTACGACAACTTCAGAAGCAGCAACAAGTACAGCAGCAGC

Supplementary Table 3 | Primer sequences used in this study

Construct	Direction	Primer (5´-3´)
L252H	Forward	CTCCTACCACCGGATCTGCCTGCTGCAC
	Reverse	CAGATCCGGTGGTAGGAGCCCTTGTAGT
I254T	Forward	CCTGCGGACCTGCCTGCTGCACCCTGT
	Reverse	GCAGGCAGGTCCGCAGGTAGGAGCCCT
